# Books and Magazines

**Published:** 1904-06

**Authors:** 


### Books and Magazines

OBSTETRIC AND GYNECOLOGIC NURSING. By Edward P. Davis, A. M., M. D., professor of Obstetrics in the Jefferson Medical College and in the Philadelphia Polyclinic. 12 mo. volume of 402 pages, fully illustrated. Second edition, thoroughly revised.

Philadelphia, New York and London: W. B. Saunders & Company. 1904. Polished Buckram, $1.75, net.

The usefulness of this book to the nursing profession is manifest by the fact that a second edition has been called for. It is necessary for an obstetric nurse to possess some knowledge of natural pregnancy and of its consequent diseases; and as gynecologic nursing is really a branch of surgical nursing, special training and instruction are required to meet the conditions arising. It would be well if every trained nurse possessed a copy of this book, for it certainly is of inestimable value.

---

Epilepsy and its Treatment. By William P. Spratling, M. D., Superintendent of the Craig Colony for Epileptics at Sonyea, N. Y. Handsome octavo volume of 522 pages, illustrated. Philadelphia, New York, London: W. B. Saunders & Company. 1904. Cloth, $4, net.

This work by Dr. Spratling is of unusual interest for many reasons: It is the first complete treatise on epilepsy since the appearance of Echeverria's work published over 33 years ago, and represents the practical experience of Dr. Spratling as superintendent of the Craig Colony for Epileptics at Sonyea, N. Y., during a period of ten years. The great progress made in the knowledge of epilepsy and its treatment during the past fifteen years certainly demanded an accurate and careful work which would include these latest advancements. Dr. Spratling has given us all that could be desired. Of particular interest are the chapters on the Psycologic and Medicolegal Aspects. An entire section is devoted to the all-important seizure type—Status Epilepticus; and treatment, general, educational, medical, and surgical, is discussed with wisdom, thought, and conservatism. The subject is bountifully illuminated by the citation of illustrative cases; and, indeed, for the entire work we have nothing but praise. General practitioners, as well as those especially interested in epilepsy, will find the book of great value.

---

Tuberculosis and Acute General Miliary Tuberculosis. By Dr. G. Cornet, of Berlin. Edited, with additions, by Walter B. James, M. D., professor of the Practice of Medicine in the College of Physicians and Surgeons (Columbia University), New York. Handsome octavo volume of 806 pages. Philadelphia, New York, London: W. B. Saunders & Company. 1904. Cloth, $5, net; half Morocco, $6, net.

This is the seventh volume to be issued in Saunders' American Edition of Nothnagel's Practice, and the remaining four volumes are in active preparation for early publication.

The American edition of Professor Cornet's exhaustive work appears at a time when the subject of tuberculosis has a peculiar claim upon the attention of mankind. Within a few years both professional and general public interest in the disease have taken enormous strides. In almost every civilized community societies for the prevention of tuberculosis are being organized, and these are composed not only of physicians but of laymen, while governments themselves are taking an active part in the movement. Under these circumstances, and at this time, the work is of interest to practitioners, for there is no other treatise which gives an equally clear and comprehensive view of this subject.

The article on Acute General Miliary Tuberculosis has been admirably written and gives a thoroughly clear understanding of this disease.

The importance of the chemistry of the tubercle bacillus and its bearing upon immunity have warranted a thorough treatment of this subject.

The work is complete and logically arranged, and the editor has made additions where necessary to bring it down to date.

---

Diseases of the Intestines and Peritoneum. By Dr. Hermann Nothnagel, of Vienna. The entire volume edited, with additions, by Humphrey D. Rolleston, M. D., F. R. C. P., Physician to St. George's Hospital, London, England. Octavo volume of 1032 pages, fully illustrated. Philadelphia, New York, London: W. B. Saunders & Company. 1904. Cloth, $5, net; half Morocco, $6, net.

This new volume in Saunders' American edition of Nothnagel's Practice is the eighth to be issued, and appearing within two months after the publication of the volume on tuberculosis, gives evidence that the publishers intend completing the series at an early date. This, one of the most valuable volumes in the series, is by the famous clinician Dr. Hermann Nothnagel himself, and is as exhaustive as it is practical. The distinguished editor, Dr. Humphrey D. Rolleston, of London, England, has used his pen most profusely, almost every page giving generous evidence of his careful editing. The editorial additions include sections on intestinal sand, sprue, ulcerative colitis, and idiopathic dilation of the colon. Appendicitis and peritonitis have been given unusual space, treatment and diagnosis receiving exhaustive consideration. The section on Intussusception has been greatly enlarged by the invaluable additions of D'Arcy Power, of England, who has made this subject his own. There are twenty inserts of great merit.

THE MOTHER'S MANUAL. By Emelyn Lincon Coolidge, M. D., visiting physician of the Ontario Patient Department of the Babies' Hospital, New York, formerly house physician of the Babies' Hospital, New York; physician in charge of the Babies' Clinic of the Society of the Lying-In Hospital of the City of New York. Price, $1. Published by A. S. Barnes & Company, New York.

This is a practical manual which every expectant mother should read and re-read, and the physician will be saved lots of needless trouble, and the baby needless pain, if he gets the expectant mother to buy this book.

---

THE PRACTICAL APPLICATION OF THE RONTGEN RAYS IN THERAPEUTICS AND DIAGNOSIS. By William Allen Pusey, A. M., M. D., professor of Dermatology in the University of Illinois; and Eugene W. Caldwell, B. S., director of the Edward N. Gibbs X-Ray Memorial Laboratory of the University and Bellevue Hospital Medical College, New York. Handsome octavo volume of 591 pages, with 180 illustrations, nearly all clinical. W. B. Saunders & Co. 1903. Cloth, $4.50, net; sheep or half Morocco, $5.50, net.

It has been the aim of the authors of this work to elucidate fully the practical aspects of the subject. It is evident that all the authentic literature which has developed since Röntgen's wonderful dicovery has been carefully digested, this being supplemented by the extensive experience of the authors. The value of the X-rays in diagnosis has been discussed in a thoroughly practical manner, and their limitations in this field indicated. Particular attention has been devoted to the use of the X-rays in therapeutics. Nearly all of the illustrations in this section represent actual clinical subjects, and show with unusual fidelity the condition before the use of the X-rays, at various stages of their application, and, finally, the therapeutic results obtained. Full details are also given as to the use and management of the apparatus necessary for X-ray work. All the methods with which the best results have been achieved have been carefully described in a comprehensive way. There are chapters on X-Ray Tubes, Induction Coils and Controlling Apparatus, Static Machines, Fluoroscopy, Radiography, Photographic Materials Used in Radiography, etc. This section is also fully illustrated with instructive photographs and drawings of the apparatus, including four beautiful full-paged colored plates of X-ray tubes. In fact, the work will be found of invaluable assistance, not only to the general practitioner, but also to the dermatologist, presenting, as it does, the very latest advances in X-ray therapeutics and diagnosis.

Hughes' Treatise on Neurology.—The Neurological Practice of Medicine; a cursory course of selected lectures in Neurology, Neuriatry, Psychology and Psychiatry; applicable to general and special practice; with 177 illustrations; after the author's class-room methods as a teacher of students. Designed for students and general practitioners of medicine and surgery. By Charles H. Hughes, M. D., president of the faculty and professor of Neurology, psychiatry and electrotherapy, Barnes Medical College; former major and surgeon-in-chief of Schofield, Winter, Hickory Street, and McDowell's College Military Hospitals; superintendent Missouri State Insane Hospital, acting and honorary member of many home and foreign medical and scientific societies, etc., etc.; member governing board of Centenary Hospital, ex-member board of health and consultant of City Hospital, Insane Hospital, etc. Price, $3. Hughes & Company, publishers, 418 N. 3d St., St. Louis. 1903.

The Neurological Practice of Medicine is a cursory course of selected lectures, from an eminent source of clinical and lecturing experience, on the essential features of neurology, neuriatry, psychology, and psychiatry, applicable to the general and special practice of medicine. The book is plainly and forcibly written in the author's well-known impressive and succinct style. Many of the pages of the book are peculiarly fascinating and eloquent, as well as accurately descriptive and scientific.

The style of the author in the amphitheater reappears in this remarkable book, as those who have sat under his clear and original teachings will discover in the reading of the several chapters.

The fruitful results of thirty years of extensive clinical experience over a portion of the vast fields of neurology and psychiatry are presented in this valuable book.

---

A Text-Book of Pathology. By Alfred Stengel, M. D., professor of Clinical Medicine in the University of Pennsylvania. Octavo volume of 933 pages, with 394 text-illustrations, many in colors, and seven full-page colored plates. Philadelphia, New York, London: W. B. Saunders & Company. 1903. Cloth, $5, net; sheep or half Morocco, $6, net.

In this work the practical application of pathologic facts to clinical medicine is considered more fully than is customary in works on pathology. While the subject of pathology is treated in the broadest way consistent with the size of the book, a successful effort has been made to present the subject from a clinician's point of view. In the second part of the work, the pathology of individual organs and tissues is treated systematically and quite fully under subheadings that clearly indicate the subject matter to be found on each page. In this edition the section dealing with General

Pathology has naturally received the greatest care and the most extensive revision. Several of the important chapters have been practically rewritten. Among the subjects that have received the greatest revision are: Ehrlich's Theology of Immunity and allied processes; Inflammation; The Bacterial Diseases, including Typhoid fever, Tuberculosis, Yellow Fever, and Dysentery; and Diseases of the Blood. In the second part of the book, that treating on Special Pathology—the revision has also been considerable, so that this part likewise represents the latest advances in the subject of Pathology. A very useful addition to the book is that of an appendix, treating of the Technic of Pathologic Methods, and giving briefly the most important methods at present in use for the study of pathology; including, however, only those methods that are unquestionably practicable. Many new illustrations, including ten excellent plates, have also been added, and some of the old replaced by new ones. We specially recommend the book to students and practitioners, as we believe it is the best we have seen.

---

THE TREATMENT OF FRACTURES: WITH NOTES UPON A FEW COMMON DISLOCATIONS. By Chas. L. Scudder, M. D., surgeon to the Massachusetts General Hospital. Fourth edition, thoroughly revised, enlarged and reset. Octavo volume of 534 pages, with nearly 700 original illustrations. Philadelphia, New York, London: W. B. Saunders & Company. 1903. Polished buckram, $5, net; sheep or half Morocco, $6, net.

Four large editions of this work in less than four years testify to its value. The book is intended to serve as a guide to the practitioner and student in the treatment of fractures of bones. The student sees the actual conditions as they exist in fractured bones, and is encouraged to determine for himself how to meet the conditions found in each individual case. Methods of treatment are described in minute detail, and the reader is not only told, but is *shown* how to apply apparatus, for as far as possible, all the details are illustrated. This elaborate and complete series of illustrations constitutes a feature of the book. There are 688 of them, all from new and original drawings and reproduced in the highest style of art. Several chapters of special importance are those on Gunshot Fractures of Bone; the Röntgen Ray and Its Relation to Fractures; The Employment of Plaster-of-Paris, and the Ambulatory Treatment of Fractures.

In this fourth edition many new illustrations have been added, thus increasing the accuracy of this part of the work. The text has been thoroughly revised, thereby bringing the book absolutely abreast the times. X-ray plates of the epiphyses at different ages have been arranged. These will be found of value not only as an anatomical study, but in the appreciation of epiphyseal lesions. An important addition is that of a chapter upon a few Common Dislocations. This chapter, like the rest of the book, is amply illustrated, and the accepted methods of treatment described.

---

THE AMERICAN YEAR-BOOK OF MEDICINE AND SURGERY FOR 1904. —A yearly digest of scientific progress and authoritative opinion in all branches of medicine and surgery, drawn from journals, monographs, and text-books of the leading American and foreign authors and investigators. Arranged, with critical editorial comments, by eminent American specialists, under the editorial charge of George M. Gould, A. M., M. D. In two volumes. Volume I, including *General Medicine.* Octavo, 673 pages, fully illustrated; Volume II, *General Surgery.* Octavo, 680 pages, fully illustrated. Philadelphia, New York, London: W. B. Saunders & Company. 1904. Per volume: Cloth, $3, net; half, Morocco, $3.75, net.

The American Year-Book of Medicine and Surgery continues to maintain its high place among works of its class. Indeed, the issue of 1904, now before us, if anything, is even better than the excellent issues of previous years. Such a distinguished corps of collaborators which the editor, Dr. George M. Gould, has enlisted as his assistants is sufficient guarantee that the essential points of progress are brought out, and the collaborators' notes and commentations are excellent. In the illustrative feature the 1904 issue fully maintains its reputation, there being fourteen full-page insert plates, besides a number of excellent text-cuts. We pronounce Saunders' Year-Book for 1904 the best work of its kind on the market, as it has always been.

---

A TEXT-BOOK OF THE PRACTICE OF MEDICINE.—By James M. Anders, M. D., Ph. D., LL. D., professor of the Practice of Medicine and of Clinical Medicine, Medico-Chirurgical College, Philadelphia. Sixth edition, thoroughly revised. Handsome octavo volume of 1300 pages, fully illustrated. Philadelphia, New York, London: W. B. Saunders & Company. 1903. Cloth, $5.50, net; sheep or half Morocco, $6.50, net.

This is the sixth edition of this unexcelled work in as many years. Such a sale can not but be a gratification alike to the author and to the publishers. In this edition the general plan and principles of classification adopted in the previous editions have been preserved. The many tabular presentations of points in differential diagnosis have been retained. Differential diagnosis is a most important branch of diagnostics, and than this tabular method we know of no superior way of familiarizing the practitioner and the student with the outstanding features of simulating diseases. Malaria, yellow fever, bacillary dysentery, cholecystitis, certain animal parasitic diseases, and the use of the X-rays in diagnosis and treatment have been fully discussed, incorporating the results of the most recent investigations. Among the new subjects introduced are Paratyphoid Fever, the Fourth Disease, Trypanosomiasis Orthostatic, Albuminuria, Transcortical Aphasia, Adiposis Dolorosa, and Amaurotic Family Idiocy. Every affection has been treated separately, particular attention being paid to its clinical character, diagnosis, and treatment. Evidently an immense mass of literature has been thoroughly digested, no pains having been spared to bring the entire work down to date, giving special reference to the daily needs of practitioners and students.

In recommending it, we believe we are recommending the best text-book on the practice of medicine on the market.

---

A Text-Book of Legal Medicine and Toxicology. Edited by Frederick Peterson, M. D., Chief of Clinic, Nervous Department of the College of Physicians and Surgeons, New York; and Walter S. Haines, M. D., Professor of Chemistry, Pharmacy, and Toxicology, Rush Medical College, in affiliation with the University of Chicago. Two imperial octavo volumes of about 750 pages each, fully illustrated. Philadelphia, New York, London: W. B. Saunders & Company. 1903. Per volume: Cloth, $5, net; sheep or half Morocco, $6, net.

This work presents to the medical and legal professions a comprehensive survey of forensic medicine and toxicology in moderate compass.

For convenience of reference the treatise has been divided into two sections—Part I and Part II—the latter being devoted to Toxicology and all other portions of legal medicine in which laboratory investigation is an essential feature. Under "Expert Evidence" not only is advice given to medical experts, but suggestions are also made to attorneys as to the best methods of obtaining the desired information from the witness. The Bertillon and Greenleaf-Smart systems of identification are concisely and intelligently described, and the advantages of each stated. An interesting and important chapter is that on "The Destruction and Attempted Destruction of the Human Body by Fire and Chemicals;" for on the determination of the human or animal source of the remains frequently depends the legal conduct of a given case, and the guilt or innocence of the accused. A chapter not usually found in works on Legal Medicine, though of far more than passing significance to both the medical expert and the attorney, is that on the medicolegal relations of the X-rays. The responsibility of pharmacists in the compounding of prescriptions, in the selling of poisons, in substituting drugs other than those prescribed, etc., furnishes a chapter of the greatest interest to everyone concerned with questions of medical jurisprudence. Also included in the work is the enumeration of the laws of the various States relating to the commitment and retention of the insane. In fact, the entire work is overflowing with matters of the utmost importance, and expresses clearly, concisely, and accurately the very latest opinions on all branches of forensic medicine and toxicology.

[A review of Vol. I appeared in a recent issue of the JOURNAL. —ED.]

---

THE FOUR EPOCHS OF WOMAN'S LIFE. Maidenhood, Marriage, Maternity, Menopause. By Anna M. Galbraith, M. D., author of "Hygiene and Physical Culture for Women." Fellow of the New York Academy of Medicine, etc. With an introductory note by John H. Musser, M. D., Professor of Clinical Medicine, University of Pennsylvania. 12mo. volume of 247 pages. Philadelphia, New York, London: W. B. Saunders & Company. 1903. Cloth, $1.50, net.

This work, written for the instruction of the laity on subjects of which every woman should have a thorough knowledge, is indeed a timely and excellent one. The fact that a second edition has been demanded in such a short time is sufficient proof that women have at last awakened to a sense of the penalties they have paid for their ignorance of those laws of nature which govern the epochs of their lives. The language used is clear and comprehensive, yet, withal, modest, and the meaning easily grasped even by those unfamiliar with medical subjects. As a further aid a comprehensive glossary of medical terms has been appended.

In this new edition the author has made some excellent additions, viz.: A section on "The Hygiene of Puberty;" one on "Hemorrhage at the Menopause a Significant Symptom of Cancer;" and one on "The Hygiene of the Menopause." These sections make the work the very best on the subject we have seen, and physicians will be doing a real service by recommending it to their patients.

---

ATLAS OF THE EXTERNAL DISEASES OF THE EYE. By Prof. Dr. O. Haab, of Zurich. Second edition, thoroughly revised. Edited, with additions, by G. E. DeSchweinitz, A. M., M. D., Professor of Ophthalmology in the University of Pennsylvania. With 98 colored lithographic illustrations on 48 plates, and 232

pages of text. Philadelphia, New York, London: W. B. Saunders & Company. 1903. Price, $3, net.

This Atlas on External Diseases of the Eye forms an excellent compaion-book to Professor Haab's "Atlas of Ophthalmology and Ophthalmoscopic Diagnosis," and is just what might be expected from an author of such broad clinical experience and trained observation. Starting with examination of the eye the student is easily and gradually led from one examination to another, thus becoming familiar with the best methods of investigating the eye for the detection of disease. In the chapters on diseases of the eye which follow, the most important diseases are clearly described and the best therapeutic measures recorded. The text has been amply illustrated by a series of beautiful chromo-lithographic plates, to each one of which a clinical history is appended. This second edition has been thoroughly revised and brought down to date, and a number of new chromo-lithographic plates added. As in the first edition, valuable editorial comments are introduced, and reference made to many of the modern therapeutic agents.

---

MORPHINISM AND NARCOMANIA from Opium, Cocaine, Ether, Chloral, Chloroform, and other Narcotic Drugs; also the Etiology, Treatment, and Medicolegal Relations. By T. D. Crothers, M. D., Superintendent of Walnut Lodge Hospital, Conn.; Professor of Mental and Nervous Diseases, New York School of Clinical Medicine, etc. Handsome 12mo of 351 pages. Philadelphia and London: W. B. Saunders & Co. 1902. Cloth, $2, net.

The alarming increase, in the last few years, of morphomania and the associated various narcomanias imperatively demands immediate attention by the medical profession. Every year the increasing prominence of this psychosis calls for more exact studies, with a fuller recognition of the conditions and causes of the disease. Medicolegally, questions of responsibility have been asked with increasing frequency, and there has been no literature and no study of the subject to afford an intelligent answer until this present volume was initiated.

The special object of this work has been to group the general facts and outline some of the causes and symptoms common to most cases, and to suggest general methods of treatment and prevention. The object could not have been better accomplished. The work gives a general preliminary survey of this new field of psychopathy, and points out the possibilities from a larger and more accurate knowledge, and so indicates degrees of curability at present unknown. The author shows his absolute familiarity with his subject in the clear, concise, and in every way admirable work which he has given to the profession, whom he has placed under merited obligations.

